# Intravenous Dexmedetomidine Administration Prior Anesthesia Induction With Propofol at 4°C Attenuates Propofol Injection Pain: A Double-Blind, Randomized, Placebo-Controlled Trial

**DOI:** 10.3389/fmed.2021.590465

**Published:** 2021-05-07

**Authors:** Yayun Lu, Yaping Gu, Lihua Liu, Xuefeng Tang, Qing Xia, Zhiyue Xu

**Affiliations:** ^1^Center of Gastrointestinal Endoscopy, Huadong Sanatorium, Wuxi, China; ^2^Department of Anesthesiology, Hospital of Stomatology, Sun Yat-Sen University, Guangzhou, China

**Keywords:** dexmedetomidine, propofol, injection pain, lidocaine, cold temperature

## Abstract

**Background:** Propofol injection pain, despite various interventions, still occurs during the anesthesia induction and causes intense discomfort and anxiety in patients. This study aimed to explore the effect of intravenous dexmedetomidine on propofol injection pain prior to anesthesia induction with propofol at 4°C.

**Methods:** A total of 251 patients (American Society of Anesthesiologists I–II) who underwent oral and maxillofacial surgery were randomly assigned to a combination group (*n* = 63), lidocaine group (*n* = 62), dexmedetomidine group (*n* = 63), and placebo-control group (*n* = 63); they received 0.5 ug/kg dexmedetomidine prior to anesthesia induction with propofol at 4°C, 40 mg lidocaine, 0.5 ug/kg dexmedetomidine prior to anesthesia induction, and normal saline, respectively. Incidence of pain, pain intensity, and reaction to the pain stimulus were evaluated by using verbal categorial scoring (VCS), a numerical rating scale (NRS), and the Surgical Pleth Index (SPI), respectively. In addition, hemodynamic parameters such as heart rate (HR) and mean arterial pressure (MAP) were also measured. The VCS and NRS were evaluated at 5 s after propofol injection. In addition, SPI, HR, and MAP were evaluated at three time points (before anesthesia induction and 5 and 30 s after propofol injection).

**Results:** The incidence of pain in the combination group (51%) was significantly lower than that in the lidocaine group (71%), dexmedetomidine group (67%), or placebo-control group (94%) (*p* < 0.001). VCS and NRS scores in the combination group were also lower compared with the other three groups (*p* < 0.001), with no statistically significant differences between the lidocaine group and dexmedetomidine group (*p* > 0.05). The SPI of the combination group decreased significantly in comparison with the other three groups at 5 s after propofol injection (*F* = 96.23, *p* < 0.001) and 30 s after propofol injection (*F* = 4.46, *p* = 0.005). Further comparisons between HR and MAP revealed no significant differences across the groups (*p* > 0.05).

**Conclusion:** Because of the sedative nature of dexmedetomidine and analgesic effect of low temperature, this study showed that intravenous dexmedetomidine prior to anesthesia induction with propofol at 4°C is highly effective in attenuating the incidence and severity of pain during injection compared with lidocaine (40 mg), dexmedetomidine 0.5 ug/kg) and placebo. This approach was not associated with any anesthesia complications.

**Clinical Trial Registration:** ClinicalTrials.gov, identifier: ChiCTR-2000034663

## Introduction

Propofol, a common intravenous anesthetic, is extensively used in induction, sedation, and maintenance of general anesthesia because of its rapid onset and quick patient recovery (1, 2). However, pain with its injection has been identified as a troubling experience for patients. About 28–90% of patients receiving propofol injection via the dorsal hand vein suffer different levels of pain intensity (3, 4). In addition, many anesthesiologists rank the pain experienced during propofol injection pain as the seventh-worst outcome among 33 known anesthesia outcomes, based on clinical importance and frequency (5). Injection pain may influence the quality of anesthesia in patients and cause an unpleasant experience (4, 6, 7).

Several factors account for propofol injection pain. For instance, increasing evidence has demonstrated that the lipid solvent irritates the vein intima and activates a local kallikrein-kinin cascade by releasing bradykinin and inflammatory factors (8, 9), and injection pain has been shown to occur when peripheral nerve endings are directly exposed to propofol (4). Moreover, investigations of clinical factors have revealed that female patients of younger age, with a peripheral vein site (especially the dorsum of hand), are highly sensitive to injection pain (10). Current recommendations suggest propofol injection in an antecubital vein for reducing propofol injection pain; however, this is not always practical in clinical situations.

Multiple strategies such as pharmacological and non-pharmacological therapy have proved to be efficacious in attenuating injection pain. In addition, drugs including lidocaine, ketamine, magnesium sulfate, and triglycerides are commonly used as pain relievers during propofol injection (4, 5, 11, 12). In a systematic review, it was concluded that lidocaine pretreatment effectively lowered the incidence of propofol injection pain (13). It is widely acknowledged that lidocaine is an acceptable anesthetic that prevents injection pain. Nevertheless, its adverse cardiovascular and hemodynamic effects and swelling at its injection site need to be further studied (14, 15). Recent reports have shown that dexmedetomidine (Dex-) is as highly effective in attenuating propofol-induced pain as lidocaine (6, 16). On the other hand, Dex- is a highly selective alpha-2 adrenoceptor agonist and exhibits analgesic, sedative, and sympatholytic properties that regulate the incidence and intensity of propofol injection pain. Non-pharmacological interventions such as the selection of larger veins (e.g., antecubital vein), adjusting injection speed, and controlling propofol dose (7, 17) are rarely in use for unknown reasons. Previous studies illustrated that temperature of propofol affected the intensity of injection pain and indicated that heating and cooling of propofol could lower pain intensity (18–20). Of note, propofol is generally kept at 25°C, and when warmed up to room temperature or 37°C, its chemical structure or efficiency may be altered. As a result, propofol at 4°C is more likely to be effective in reducing the intensity of pain.

Compared to lidocaine used alone, combination therapy is recommended in clinical situations (3). Therefore, the purpose of our study was to explore the effect of intravenous Dex- prior to anesthesia induction with propofol at 4°C in attenuating propofol injection pain compared with lidocaine applied independently.

## Materials and Methods

### Study Design and Ethical Aspects

This study was designed as a double-blinded, randomized, and placebo-controlled clinical trial and all patients underwent oral and maxillofacial surgery at the Affiliated Stomatological Hospital of Sun Yat-sen University. The objectives and procedures of this study and adverse effects were explained completely, and all patients signed the written informed consent after a preoperative interview with an anesthetist. This clinical trial was conducted in accordance with the Declaration of Helsinki and obtained approval by the Ethics and Research Committee of the Affiliated Stomatological Hospital of Sun Yat-sen University, Guangzhou, China [Number (2018)3-105]. This trial was registered at the Chinese clinical trial registry (http://www.chictr.org.cn. Number ChiCTR-2000034663).

### Study Participants

**Figure 1:**
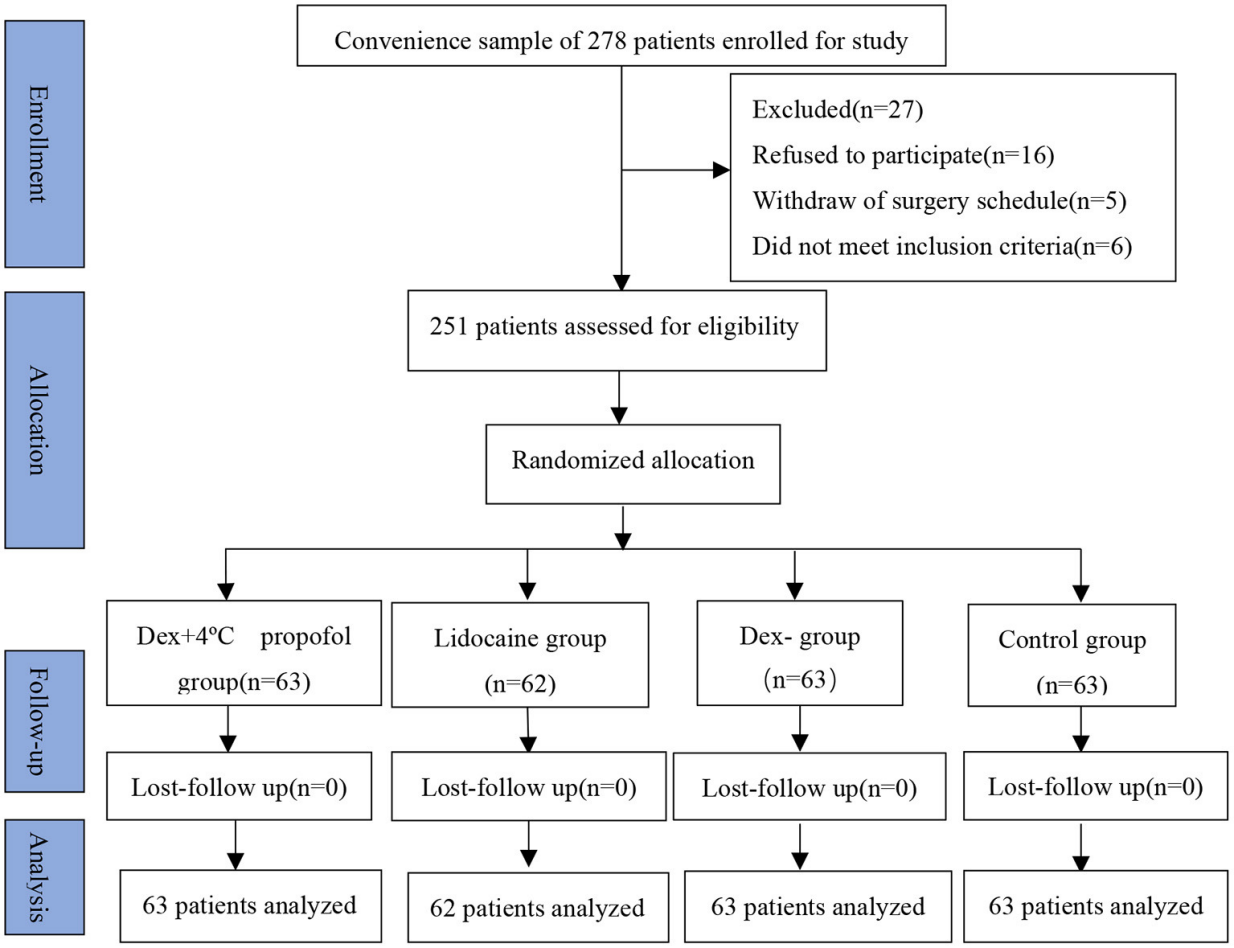
CONSORT flow diagram of the study.

All patients were scheduled for elective surgery under general anesthesia between September 2018 and July 2019. The main exclusion criteria were as follows: patients aged <18 years, status of ASA > II, history of any drug allergies or use of analgesic medication within 24 h before surgery, any organ dysfunction, and neurological disease and mental disorders. A total of 278 patients were initially enrolled in this study; however, 16 patients were excluded after they declined to participate or surgery was canceled, 5 patients were also excluded as a result of the withdraw of surgery schedule, and 6 patients did not meet the inclusion criteria. Eventually, 251 patients aged between 18 and 56 years with the American Society of Anesthesiologists physical status I–II (ASA I–II) were eventually enrolled in the four groups through random allocation (Figure 1).

### Randomization and Blinding

Using a computer-generated randomization table, patients were randomly assigned into “Dex- (0.5 ug/kg) plus propofol at 4°C group (combination group),” “lidocaine group (40 mg),” “Dex- (0.5 ug/kg),” and “placebo-control group” according to a 1:1:1 ratio. A random allocation sequence of 251 consecutive and numbered envelopes, representing different group assignments, were directly delivered to an anesthetic nurse. Throughout the period of study and data collection, the principal investigator and participants remained unaware of each group assignment, while two anesthetic nurses and anesthetists were also blinded to participants' group assignment. the group assignments were announced only after finishing data analysis.

### The Study Protocol and Data Collection

Demographic data including age, gender, American Society of Anesthesiologists (ASA) physical status, weight, and height were recorded for all patients in the 30 min before they entered the operating room. After entering the operating room, patients received standard monitoring of the electrocardiograph, non-invasive blood pressure, and pulse oximetry. Hemodynamic indexes, including mean arterial pressure (MAP) and heart rate (HR), were recorded when patients remained silent. After that, an 18G cannula was inserted into a large vein on the dorsum of the non-dominant hand 20 min before anesthesia induction, and an infusion of lactated Ringer′s solution (5 ml/kg/h) was applied to maintain its patency.

Prior to anesthesia induction, Dex- (200 ug/2 ml, Hengrui Medicine, Jiangsu, China) was dissolved in a 50-ml syringe and the combination group was injected of 0.5 ug/kg DEX- for about 5 min, 40 mg lidocaine was dissolved in a 10-ml syringe and gently injected in 3 min in the lidocaine group, and patients in the placebo-control group were informed of being injected with an analgesic and received an equivalent volume of normal saline. Injections of Dex-, lidocaine, and normal saline were all undertaken via a three-way connector. Then, 6 L/min of pre-oxygenation was administered to patients with a mask, after which the principal anesthetist informed the anesthetic nurse to start anesthesia induction. During anesthesia induction, the combination group received propofol refrigerated at 4°C, and another three groups received propofol at room temperature. Because propofol injection (0.5 mg/kg) is a targeted dose, it should be injected within 30 s. However, it is worth noting that injection of 4°C propofol must be close to the puncture point of the intravenous infusion to avoid increasing its temperature. Therefore, another three-way connector close to the intravenous site was used to inject 4°C propofol.

In this experiment, primary outcomes included data collection on the pain scale of VCS, NRS, and SPI, and MAP and HR were recorded as secondary outcomes. Two anesthetic nurses assessed the intensity of propofol injection pain in about 5–10 s by using VCS and NRS. VCS is a 4-point pain scale: 0–no pain, 1–mild pain with no behavioral sign, 2–moderate pain accompanied by a behavioral sign, and 3–severe pain with a strong vocal response accompanied by facial grimacing, arm withdrawal, or crying (7). Patients were instructed to report any discomfort through an 11-point NRS which ranges from 0 (no pain) to 10 (worst pain) (15). The arms of all patients were covered with a cloth to blind the two nurses to the group assignment. Moreover, a measure of the SPI was taken via an anesthesia monitor that recorded the patient's response to stressful surgical stimuli or pain during general anesthesia. MAP, HR, and SPI were separately recorded at three time points: before propofol injection, 5 s after propofol injection, and 30 s after propofol injection. After the targeted 0.5 mg/kg of propofol was finished, the remaining induction of propofol (1.5 mg/kg) at room temperature was administered immediately, accompanied by a complete injection of cis-atracurium (0.2 mg/kg) and remifentanil (1 ug/kg). After patients' eyelash reflexes began to disappear, a mixture of 100% oxygen and air combined with sevoflurane was delivered to the patient via a face mask. In the meantime, an anesthesiologist checked any anesthesia-induced complications after tracheal intubation. The dose of propofol for anesthesia induction, duration of surgery, and recovery time were recorded for the four groups.

### Sample Size

Based on findings from a previous study in which 64% of patients experienced propofol injection pain (13), it was hypothesized that pre-administration of Dex- plus 4°C propofol would cause a 40% reduction in the injection pain. In our study, to provide the two-sided test at an α level of 0.05 and a power of 80%, we used a minimum sample size of 54 patients per group to detect a significant difference, considering a 15% dropout rate. We finally enrolled 63 patients in the combination group (0.5 ug/kg Dex- + 4°C propofol), 62 patients in the lidocaine group, 63 patients in the Dex- group and 63 patients in the placebo-control group (normal saline).

### Statistical Analyses

All data were analyzed using SPSS 20.0 for Windows (SPSS, Inc, Chicago, IL, USA). Demographic and clinical characteristics were expressed as means ± standard deviation (continuous variables) or frequency (categorical variables) and analyzed by the χ^2^-test and one-way ANOVA test across the groups. Because the VCS scores were not normally distributed, we used the Kruskal-Wallis test to compare VCS, whereas the Mann-Whitney *U*-test was used for multiple comparisons between different groups. Comparisons of the incidence of pain between groups were determined by the χ^2^-test or Fisher's exact test. Repeated measures ANOVA was used to compare hemodynamic parameters (HR, MAP) between different groups and the three time points. A comparison of the two groups was made by Bonferroni correction where necessary. A *p* < 0.05 was considered statistically significant.

## Results

### Demographic Characteristics

Demographic characteristics and clinical baseline data are summarized in Table 1. No significant differences were found between the four groups regarding age, gender, status of ASA, height, weight, HR, MAP, SPI, recovery time, and dose of propofol for anesthesia induction (*p* > 0.05).

**Table 1:** Baseline characteristics of included patients (*n* = 251).

**Variables**	**Dex+4^**°**^Cpropofol (*n =* 63)**	**Lidocaine pre-administration (*n =* 62)**	**Dex- pre-administration (*n =* 63)**	**Control (*n =* 63)**	***F*,χ^2^**	***p***
	**Mean ± SD**					
**Age**	31.32 ± 8.69	31.74 ± 7.97	32.76 ± 8.85	32.14 ± 9.62	0.33*^*F*^*	0.81
**Gender**						
Female Male	34 29	31 31	31 32	28 35	1.16*^χ^2*	0.76
**ASA**					5.98*^χ^2*	0.11
I II	53 10	49 13	47 16	57 6		
**Height(m)**	1.68 ± 0.08	1.67 ± 0.08	1.70 ± 0.08	1.66 ± 0.07	2.47*^F^*	0.06
**Weight(kg)**	57.59 ± 10.02	60.11 ± 10.43	56.78 ± 9.19	59.06 ± 8.82	1.49*^F^*	0.22
**Heart rate(bpm)**	75.81 ± 11.38	74.60 ± 10.80	74.33 ± 10.12	77.33 ± 12.29	0.94*^F^*	0.42
**Mean arteria pressure(mmHg)**	84.59 ± 8.48	83.05 ± 8.44	82.76 ± 8.02	83.54 ± 8.27	0.59*^F^*	0.62
**Surgical pleth index (SPI)**	69.27 ± 11.20	68.34 ± 9.78	69.90 ± 10.29	68.37 ± 11.21	1.25*^H^*	0.74
**Recovery time(sec)**	285.84 ± 42.78	273.13 ± 42.14	282.48 ± 41.91	280.42 ± 36.73	3.02*^H^*	0.39
**Dose of propofol for anesthesia induction(mg)**	115.17 ± 20.05	120.23 ± 20.86	113.56 ± 18.39	118.11 ± 17.64	4.02*^H^*	0.26

*F, ANOVA statistics; χ2, Chi-square test statistics; H, Kruskal-Wallis test statistics*.

### Comparisons Between Groups in the Incidence and Severity of Propofol Injection Pain

The total incidence of propofol injection pain in the combination group (51%) was significantly lower than in the lidocaine group (71%), Dex- group (67%), or control group (94%) (*p* < 0.001) (Table 2). According to the different levels of severity of pain among the four groups, the incidence of patients with no pain in the combination group (49%) was the highest compared with the placebo-control group (6%), the lidocaine group (29%), and Dex- group (33%) (*p* < 0.001). Additionally, no significant differences in mild pain were found among the groups. However, the incidence of moderate pain in the placebo-control group (40%) was significantly higher compared to the other three groups (*p* < 0.001). Further, severe injection pain occurred in only three patients in the placebo-control group.

**Table 2:** Incidence and severity of propofol injection pain (*n* = 251).

**Severity of pain** **(VCS)**	**Dex-+4°C propofol group (*n =* 63)**	**Lidocaine group (*n =* 62)**	**Dex- group (*n =* 63)**	**Control group (*n =* 63)**
No pain (0)	31(49%)^*^^#^^&^	18(29%)^&^	21(33%)^&^	4(6%)
Mild pain (1)	29(46%)	35(56%)	29(46%)	31(49%)
Moderate pain (2)	3(5%)^*^^#^^&^	9(15%)^&^	13(21%)^&^	25(40%)
Severe pain (3)	0	0	0	3(5%)
Total Pain	32(51%)^*^^#^^&^	44(71%)^&^	42(67%)^&^	59(94%)

* *p < 0.001; compared with lidocaine group*.

# *p < 0.001; compared with Dex- group*.

& *p < 0.001; compared with the control group*.

### Comparisons Between Groups in the Scores of Pain VCS, Pain NRS, and SPI

By comparing the pain scores of VCS, NRS, and SPI between groups (Table 3), we found significant differences in VCS scores (*H* = 44.90, *p* < 0.001), Also, the NRS scores had statistically significant differences among the four groups (*F* = 34.71, *p* < 0.001). Meanwhile, it was obvious that scores of VCS and NRS in the combination group were also lower compared with the other three groups (*p* < 0.05). In other words, the combination group was superior to the other three groups with respect to pain reduction. Through ANOVA, SPI showed a significant difference among groups in 5 s after propofol injection (*F* = 96.23, *p* < 0.001) and 30 s after propofol injection (*F* = 4.46, *p* = 0.005). Primary outcomes in the combination group were significantly different from those in the other three groups, whereas the lidocaine group and Dex- group showed a significant difference compared with the placebo-control group.

**Table 3:** Comparisons of pain scores (VCS/NRS) and surgical pleth index between groups (*n* = 251).

**Variable**	**Time**	**Dex+4°C propofol (*n =* 63)**	**Lidocaine pre-administration (*n =* 62)**	**Dex- pre-administration (*n =* 63)**	**Control (*n =* 63)**	***F*,χ^2^**	***p***
**Mean ± SD**							
Scores of VCS	5 s after propofol injection	0.56 ± 0.59^#^^&^^**^	0.85 ± 0.65^*^	0.87 ± 0.73^*^	1.43 ± 0.69	44.67*^H^*	<0.001
Scores of NRS	5 s after propofol injection	2.86 ± 1.35^#^^&^^**^	3.61 ± 1.70^*^	3.57 ± 1.96^*^	5.27 ± 1.88	21.72*^F^*	<0.001
Surgical pleth index (SPI)	Prior to anesthesia induction	69.27 ± 11.20	68.34 ± 9.78	69.90 ± 10.29	68.37 ± 11.21	0.32*^F^*	0.81
	5 s after propofol injection	53.38 ± 9.93^&^^**^	57.77 ± 9.53^&^^*^	58.89 ± 7.26^&^^*^	77.60 ± 7.78	96.23*^F^*	<0.001
	30s after propofol injection	39.35 ± 7.05^#^^&^^*^	43.37 ± 8.97	42.89 ± 8.17	44.32 ± 8.27	4.46*^F^*	0.005

* *p < 0.05;*

** *p < 0.001;*

# *: compared with lidocaine group*;

& *, compared with Dex- group;*

*^$^, compared with control group; F, ANOVA statistics; H, Kruskal-Wallis test statistics*.

### Comparisons Between Groups in the HR and MAP

Comparisons of the hemodynamic parameters (HR, MAP) among the four groups are presented in Table 4. Repeated-measures ANOVA for hemodynamic parameters revealed a significant difference at different time points (HR, *F* = 186.28, *p* < 0.001), (MAP, *F* = 182.23, *p* < 0.001). Further, comparisons between HR and MAP showed no significant difference among the four groups (HR, *F* = 1.24, *p* = 0.27), (MAP, *F* = 1.05, *p* = 0.31), and interaction by times and groups was also showed no significant difference (HR, *F* = 0.83, *p* = 0.37), (MAP, *F* = 0.69, *p* = 0.41).

**Table 4:** Comparisons of Hemodynamic parameters between groups (*n* = 251).

**Variables**	**Time**	**Dex+4°C propofol (*n =* 63)**	**Lidocaine pre-administration (*n =* 62)**	**Dex- pre-administration** **(*n =* 63)**	**Control (*n =* 63)**	***Source***	***F***	***p***
**Mean ± SD**								
HR(bpm)	Prior to anesthesia induction	75.81 ± 11.38	74.60 ± 10.80	74.33 ± 10.12	77.33 ± 12.29	Time Group	186.28 1.24	<0.001 0.27
	5 s after propofol injection	76.16 ± 11.69	75.71 ± 8.77	75.29 ± 9.22	79.14 ± 11.73	T*G	0.83	0.37
	30 s after propofol injection	67.81 ± 7.95	66.60 ± 6.00	67.40 ± 7.99	68.75 ± 8.71			
MAP(bpm)	Prior to anesthesia induction	84.59 ± 8.48	83.05 ± 8.44	82.76 ± 8.02	83.54 ± 8.27	Time Group	182.33 1.05	<0.001 0.31
	5 s after propofol injection	80.19 ± 8.36	78.84 ± 8.57	78.83 ± 6.80	82.05 ± 8.51	T*G	0.69	0.41
	30 s after propofol injection	66.84 ± 7.38	68.24 ± 6.12	66.35 ± 6.45	69.14 ± 7.61			

*HR, Heart rate; MAP, Mean arterial pressure; F, Repeated Measures ANOVA*.

## Discussion

We designed a randomized, double-blind study to explore the efficacy of 0.5 ug/kg Dex- given prior to anesthesia induction with propofol at 4°C to attenuate injection pain in patients under general anesthesia. According to the findings, it was evident that 0.5 ug/kg Dex- pre-administration plus 4°C propofol had a favorable effect in alleviating propofol injection pain compared to lidocaine (40 mg) and Dex-(0.5 ug/kg). In addition, the difference between HR and MAP was not statistically significant among the four groups, and no anesthesia-induced complications such as anaphylaxis, hypotension, and bradycardia occurred during surgery.

Prevention of propofol injection pain deserves more attention, especially because of the discomfort experienced during anesthesia induction, and the mechanism of propofol injection pain should be elucidated. It is well-recognized that propofol is an excellent anesthetic that has a phenol group, and resultant pain can occur immediately or be delayed after propofol injection. However, propofol can directly irritate the afferent nerve endings within the mucous membranes and venous intima and cause immediate pain. Additionally, delayed pain arises when the kallikrein-kinin systems are activated by propofol molecules, generating bradykinin and inducing local vasodilation and hyperpermeability (6). Consequently, the contact between the aqueous phase of propofol and free nerve endings of vessel walls is increased (21). Nevertheless, a previous study revealed that the generation of bradykinin via activation of the plasma kallikrein-kinin system is completely not associated with propofol injection pain (22). It is worth noting that immediate pain caused by direct contact between propofol molecules and peripheral nerve endings is predominantly linked to propofol injection pain. As a result, we evaluated pain intensity in 5 s after injection of propofol by using VCS and NRS scales. Because propofol injection pain occurs rapidly once propofol injection starts, premedication for injection pain prior to anesthesia induction is a common approach in clinical practice.

Because of its local anesthetic effects on the venous intima by inhibiting sodium-specific ion channels and stabilizing the kinin cascade (23), lidocaine pretreatment is widely used to produce a relative reduction in propofol injection pain. To achieve continuous effects with the use of lidocaine in reducing propofol injection pain, a tourniquet applied to the forearm is necessary to maintain its local analgesic effect by venous occlusion. However, the application time of the tourniquet should last about 30–60 s to exert a maximal effect, which unfortunately causes tourniquet-induced pain or discomfort, and the failure rate of lidocaine in pain relief ranged from 13 to 40% (18). In our study, we used a dose of 40 mg in the lidocaine group as previously recommended in a meta-analysis by Picard and Tramer (24). Given the discomfort caused by a tourniquet, lidocaine was slowly injected without a tourniquet in this trial. Nevertheless, a recent study used a mixture of lidocaine and propofol to pretreat injection pain, and it did not alleviate the propofol injection pain (25). In our study, the incidence of injection pain was reduced from 94% (control group) to 71% (40 mg lidocaine group), which suggested lidocaine pre-administration prior to anesthesia is effective in pain relief. However, the incidence of injection pain in the lidocaine group was still high, even though most patients experienced mild pain intensity.

Dex- may have a great impact on pain relief because of its significant sedative action. Dex-, as a highly potent alpha-2 adrenoreceptor agonist, has been proved to exert significant analgesic effects by raising the pain threshold and is also widely used in the sedation of critical patients on the basis of its sedative and anti-anxiety properties. The analgesic effect of Dex- could be achieved with a high dose of 1 ug/kg over 10 min (26); however, to avoid anesthetic complications, our study used just 0.5 ug/kg of Dex- pre-anesthesia, and its effect on pain relief possibly originates from its sedation property. Current evidence suggests that Dex- acts by inhibiting the release of substance P from the dorsal horn of the spinal cord and the spinal ERK1/2 signaling pathway (27–29). A study by Park et al. demonstrated that Dex- exerted a dose-dependent analgesic effect in rat models. Elsewhere, it was revealed that a high dose of Dex- in patients resulted in rapid pain relief (30, 31). Based on a previous report, a common dose of Dex- used in premedication of propofol injection pain was 0.25, 0.5, or 1 ug/kg; however, 0.25 ug/kg of Dex- did not effectively alleviate propofol injection pain (32). A recent study showed that the safety properties of Dex- at doses of 0.5 or 1 ug/kg could stabilize hemodynamics (33). Upon consultation with the anesthetist, we considered a 0.5 ug/kg dose of Dex- to be the most appropriate. Our study also indicated that there was no statistically significant difference between Dex- and lidocaine in reducing propofol injection pain, and were similar to prior finding (6). Hence, the intravenous administration of Dex- appeared to be effective in pain relief as lidocaine.

Furthermore, low-temperature propofol generated contradictory outcomes in reducing injection pain. However, previous studies showed that using cold propofol in pain relief is an effective method because of decreased speed of the kinin cascade and the stabilization of local pain mediators (20, 34). On the contrary, findings from other studies have revealed that cold propofol had no impact on pain reduction (35), although pre-administration of 4°C propofol was recently shown to potentially lower the incidence of propofol injection pain from 70 to 30%. However, a systematic review demonstrated that 4°C propofol was not effective in reducing injection pain (*RR* = 0.82, 95% CI:0.64–1.04) (18). Therefore, a single use of 4°C propofol may not result in a remarkable analgesic effect. Therefore, we considered the analgesic effect of combining 0.5 ug/kg Dex- prior to the induction with propofol at 4°C. Notably, results showed that the incidence of injection pain was reduced from 94% (control group) to 51% (combination group), which explained the potential cumulative analgesic effect of 0.5 ug/kg Dex- in combination with propofol at 4°C.

There are some limitations to our study. First, we did not use a high dose of Dex-, such as 0.75 or 1 ug/kg, so it is not clear whether the effects of a high dose of dexmedetomidine could significantly reduce pain. Second, patients were not followed up for potential adverse effects after discharge of operation room. Finally, because patients in the lidocaine group did not use a tourniquet for venous occlusion, the contact between lidocaine and venous intima was not efficient. This explains why pain alleviation in the lidocaine group was inferior to that of the combination group.

## Conclusion

In summary, considering the sedative nature of Dex- and the analgesic effect of low temperature, this double-blind, randomized, placebo-controlled trial demonstrated that intravenous Dex- prior to anesthesia induction with propofol at 4°C can effectively attenuate propofol injection pain compared with lidocaine (40 mg), Dex- (0.5 ug/kg), and placebo. It is worth noting that no significant adverse events arose during the intervention process.

## Data Availability Statement

The datasets presented in this study can be found in online repositories. The names of the repository/repositories and accession number(s) can be found at: https://pan.baidu.com/s/1_tLnhB22hZVe4XbDPlHQSA~Code:7jrm&lt.

## Ethics Statement

The studies involving human participants were reviewed and approved by Ethics and Research Committee of the Affiliated Stomatological Hospital of Sun Yat-sen University, Guangzhou, China. The patients/participants provided their written informed consent to participate in this study.

## Author Contributions

YL, YG, and ZX conceived of the study and improved the design. YL and LL were responsible for collecting clinical data. XT and QX provided statistical analysis and interpretation. All authors approved the final manuscript.

## Conflict of Interest

The authors declare that the research was conducted in the absence of any commercial or financial relationships that could be construed as a potential conflict of interest.
